# Navigating a climate of administrative burden: the perspectives of young adult undocumented immigrants in applying for COVID-19 disaster relief assistance for immigrants in California

**DOI:** 10.3389/fpubh.2024.1304704

**Published:** 2024-02-15

**Authors:** Irving C. Ling, Hye Young Choi, May Sudhinaraset

**Affiliations:** ^1^Fielding School of Public Health, University of California, Los Angeles, CA, United States; ^2^School of Medicine, Yale University, New Haven, CT, United States

**Keywords:** administrative burden, DACA, mixed status families, undocumented Asian immigration, undocumented Latina/o immigration, COVID-19 relief

## Abstract

Undocumented immigrants experienced high levels of economic insecurity during the COVID-19 pandemic while being excluded from government-based relief and unemployment benefits. In April 2020, California became the first state to offer financial aid to undocumented immigrants through the innovative Disaster Relief Assistance for Immigrants (DRAI) program in collaboration with several community-based organizations (CBOs). However, the process of applying for aid was marked by many implementation challenges, such as intake and language access; however, little data exists on the direct experiences of the undocumented community. This qualitative study examines the experiences of undocumented Asian and Latinx young adults living in California in applying for DRAI through framework of administrative burden. Themes distilled from participant experiences highlight how administrative burden via learning, psychological, and compliance costs shape the ways in which undocumented immigrants navigate policies and programs, such as DRAI. These experiences highlight the need for policymakers to address structural and programmatic administrative burdens in policy development; failure to do so result in detrimental impacts that outweigh financial benefits or cause communities to forgo needed resources.

## Introduction

Undocumented communities faced economic and social challenges during the COVID-19 pandemic. However, undocumented immigrants were excluded from the vast majority of federal and state-based COVID-19-related financial relief, including Economic Impact Payments (EIP) through the 2020 Coronavirus Aid, Relief, and Economic Security Act (CARES), and unemployment benefits (1). Estimates suggest that US citizens were eligible for upwards of 20 times the amount of economic relief compared to undocumented workers between 2020 and 2021 (2). Federal aid, such as the stimulus funds distributed through the 2020 CARES Act, excluded undocumented taxpayers who filed using an individual tax ID number (ITIN) or invalid social security number (SSN). Even families who filed jointly with someone using an ITIN or invalid SSN were also barred from receiving aid, meaning that almost 5 million citizen spouses and their children in mixed status families were excluded from COVID-19 relief as well (1). Among a survey of 91,000 undocumented immigrants across California during the pandemic, 87% of respondents reported that they had unmet housing needs and over half of respondents reported food, transportation, and utility insecurity, respectively (3).

In April 2020, California became the first state to offer governmental financial relief to undocumented immigrants through the Disaster Relief Assistance for Immigrants (DRAI) program. This was an unprecedented program that provided $75 million in state-based funding administered by the California Department of Social Services (CDSS) in partnership with 12 immigrant-serving community-based organizations (CBOs) throughout California. Individuals were eligible for DRAI if they were (1) undocumented, (2) did not qualify for federal financial assistance, and (3) experienced hardship as a result of COVID-19. Eligibility was not means tested and CDSS stated that applying and receiving funding would not affect immigration status otherwise (4). The program aimed to serve 150,000 individuals with assistance of $500 per individual with a $1000 limit per household. The process of application required individuals to complete intake with their respective regional CBO to confirm eligibility and finalize aid logistics. The application for DRAI went live on May 18th, 2020 and ended on June 30th, 2020. It was widely anticipated that demand would be exceedingly high given that funding was very limited for an estimated population of over 1.5 million people (5). Multiple news outlets reported that individuals experienced long wait times, busy lines, and some were ultimately unable to complete their application; however, little data exists on the direct experiences of the undocumented community (6, 7).

In addition to facing logistical challenges in applying for DRAI aid, undocumented immigrants must also navigate these relief programs within a larger ecosystem of burden across laws, policies, and procedures. Historically, the U.S. immigration system has deployed a series of bans, quotas, and other restrictions, often around race and ethnicity, in determining who is allowed to reside in the country (8). During the Trump administration, the reversal of several federal immigration programs, including Deferred Action for Childhood Arrivals (DACA) and Temporary Protected Status (TPS), in addition to increased immigration enforcement actions contributed to climate of fear and distrust of services that remain under the Biden administration (9–11). In this climate, immigrants are deterred from accessing necessary benefits to address housing, health, and food insecurity to avoid scrutiny and enforcement, such as when changes were proposed to the definition of public charge that would have expanded the breadth of public services that, if used, that could jeopardize one’s green card status and pathway to citizenship (12, 13).

Moynihan et al. in studying the effects of burden in different areas of policy implementation developed the analytical framework of administrative burden to examine the relationships between people, politics, and power (14). Moynihan et al. posited that understanding why burdens are created and how they are distributed between the individual and the state, particularly along lines of race and other identities of difference, are central questions to the study of public administration. This analytic lens also illuminates how administrative burdens can be a powerful vehicle for “policymaking by other means,” by which immigration, for example, can be restricted by enacting exclusions on access to healthcare or allowing immigration enforcement to occur in clinics and hospitals, without actually having to pass federal immigration legislation (8, 14). Because these policy changes can operate via informal channels, such as memos or departmental guidance, administrative burden can be difficult to mitigate or undo. The Yale Immigration Policy Tracking Project has shown that more than 700 immigration actions created during the Trump Administration remain fully or partially in effect during the subsequent Biden Administration (15). In examining the perspectives of undocumented immigrants impacted by this regime of immigration policies, administrative burden also highlights the individual experience of government that is “shaped through the burdens” they encounter in their interactions with the state (14).

We build on this literature by utilizing this broad conceptualization of administrative burden that not only examines an individual’s experience of policy as onerous, but also illuminates the structural effects that policy implementation can have on a whole communities. This framework of administrative burden is comprised of three main components: (1) learning costs, or the need for individuals to learn and process information about policy, (2) psychological costs, or the stress or stigma of participating in policy, and (3) compliance costs, or the required protocols and procedures to participate in programs and the requirement to respond to policy demands (14). While the effects of immigration policy on undocumented immigrants have been studied, the lens of administrative burden remains largely underutilized outside of work from Moynihan et al.

For undocumented immigrants, surveillance, criminalization, and deportation serve as the foundation on which a regime of administrative burden has been built on. It is through this regime of burden that everyday life is subject to enforcement and immigrants must navigate risk of accessing services, which may be perceived to involve disclosure to authorities and government surveillance systems, thus risking deportation (12, 16). Within this framework, burden can be employed as an expansive, yet concise indicator of the practical impacts of policy on the daily lives of undocumented immigrants. Furthermore, we can examine how DRAI, despite being designed as a policy to relieve financial burden, can come to engender its own set of administrative burdens in relationship to the wider immigration policy ecosystem.

In the context of increased bureaucratization and marginalization, children of immigrants play key roles as brokers of services and information for their families (17). By translating, navigating, and securing resources, children of immigrants often reduce the negative impacts of exclusive policy environments on their families. However, the act of brokering is shaped by the sociopolitical traits of brokers and the policies themselves, which can give rise to additional disparities within the undocumented community (17). As such, we highlight the experiences of undocumented young adults in applying to the DRAI program on behalf of their households. To our knowledge, this paper is the first to examine how decisions to apply for aid are embedded within a larger climate of burden arising from precarious immigration status.

## Methods

### Study overview

This study uses qualitative data collected between June and October 2020. We employed a community-engaged approach in collaboration with a Community Advisory Board (CAB) including undocumented immigrants and experts in health policy, education, and immigrant advocacy. All project materials were approved by the [withheld] Institutional Review Board.

### Data collection and analysis

Twenty participants were recruited through snowball sampling. Eligible individuals were: (1) Asian or Latinx; (2) undocumented with or without DACA; (3) 18–39 years old; and (4) residing in California during the study. Eligibility by race and ethnicity was limited to Asians and Latinx, the fastest-growing and largest undocumented groups in the country, respectively (5, 18). Given that DACA is one of the most significant federal immigration policies passed in the past decade, this study focused on both those with and without DACA. The age range of young adults represents those who would be recipients of the DACA program at the time of the study. For example, per DACA requirements that were implemented in 2012, recipients could be no older than 30 years; therefore at the time of study, 39 years of age would be the maximum age of earliest potential recipients of the program (19). All participants provided verbal informed consent and received a $20 electronic gift card to join a 60-min recorded interview on Zoom. Interviews were conducted using a semi-structured interview guide approved by the Community Advisory Board. Audio recordings were transcribed via Otter.ai for coding on DeDoose, a qualitative analysis software. Two trained researchers reviewed transcripts and codes were identified in an iterative process and data saturation was reached at 20 interviews (20).

## Results

### Study demographics

Equal numbers of Latinx and Asian participants were represented in the study. This was a highly educated population with all participants having completed some form of higher education. Almost half of participants reported that they did not speak English in their households. While 40 % of the study population resided in Los Angeles County, there was representation across California in the remaining sample. All participants belonged to mixed status families, which often included undocumented, DACA-ineligible parents (Table 1).

**Table 1:** Study demographics.

Demographics (*n* = 20)	
Age, mean (SD)	27 (3.3)
Gender identity, n (%)	
Cis-woman	10 (50.0)
Cis-man	10 (50.0)
Race, n (%)	
Asian	10 (50.0)
Latinx	10 (50.0)
Languages other than English spoken*	
Chinese (Including dialects)	2 (10.0)
Korean	6 (30.0)
Portuguese	2 (10.0)
Spanish	10 (50.0)
Tagalog	2 (10.0)
Zapotec	1 (5.0)
Highest level of education completed, n (%)	
Some college or 2-year community college	3 (15.0)
4-year college or university	14 (70.0)
Graduate or professional school	3 (15.0)
English spoken at home household, n (%)	
Yes	11 (55.0)
No	9 (45.0)
County of residence, n (%)	
Alameda	2 (10.0)
Contra Costa	1 (5.0)
Los Angeles	8 (40.0)
Orange	2 (10.0)
Riverside	1 (5.0)
San Francisco	2 (10.0)
San Joaquin	1 (5.0)
Santa Clara	2 (10.0)
Santa Cruz	1 (5.0)

*These questions allowed for multiple answers.

### Awareness of disaster relief assistance for immigrants funding and program engagement

At the time of the study (June 2020 to October 2020), only a quarter of participants (*n* = 5) had successfully obtained funding with an additional two participants still awaiting funding after completing the application process. Forty percent of participants (*n* = 8) were aware of the DRAI program, but had not started or completed the application. The remaining five participants stated that they were completely unaware of the DRAI program (Table 2).

**Table 2:** COVID aid engagement.

EIP/CARES engagement, n (%)	
Personally received	8 (40.0)
Benefitted through known recipient	3 (15.0)
Did not personally receive or benefit	6 (30.0)
Decline to answer	3 (15.0)
DRAI engagement, n (%)	
Applied and received the benefit	5 (25.0)
Applied, but have not received the benefit yet	2 (10.0)
Aware, but did not start or finish applying	8 (40.0)
Unaware and did not apply	5 (25.0)
Non-governmental aid engagement, n (%)	
Applied and received the benefit	2 (10.0)
Applied, but have not received the benefit yet	2 (10.0)
Aware, but did not start or finish applying	4 (20.0)
Unaware and did not apply	11 (55.0)
Decline to Answer	1 (5.0)

### Economic need during the COVID-19 pandemic

The COVID-19 pandemic impacted economic conditions of participants and their families. Almost all participants were affected by economic insecurity. Job insecurity was the most common concern, affecting 80% (*n* = 16) of participants, who reported that they, themselves, or an immediate member of their household experienced either reduced hours or complete job loss during the COVID-19 pandemic (Table 3). Many participants reported that their family members worked in sectors, such as food and hairdressing, that were vulnerable to the effects of COVID-19 as well as pandemic-related policies, such as shelter-in-place (Table 4A.1). Given how the pandemic affected multiple industries, it often meant that several income streams for any given household were impacted simultaneously, and that potential for job recovery were similarly limited as well.

**Table 3:** Economic impacts of COVID-19 pandemic.

Reported economic impact of COVID-19 pandemic (n, %)	
Employment insecurity	16 (80.0)
Changed spending habits	10 (50.0)
Housing insecurity	8 (40.0)
Food insecurity	5 (25.0)
Transportation insecurity	4 (20.0)
Personal protective equipment insecurity	4 (20.0)

**Table 4:** Representative themes and sub-themes.

Theme	Sub-themes
Economic Need During the COVID-19 Pandemic	**Housing insecurity** “The economic impact to the family was immediate, two weeks loss of income for my dad (barber) and then half hours gone from my mom’s schedule (waitress), but we had some savings… And it was only going to last until May… and so of course that [shelter in place] was extended… the concern about whether we’d actually be able to stay in the apartment because we did not have the funding.” (Latinx participant) “My apartment complex receiving notice of like, the management still insisted on collecting rent, even after the eviction moratorium… I look up the management and the person who owns it and then found a whole lengthy history of like him… just parading Trump… and there’s like, even like a page of him on an anti-eviction mapping project… I did not want to [fight this]… [but] also considering like, ‘Okay, well, you know, mom is undocumented, you are kinda in the process of like getting your status adjusted, so yeah, no, maybe not right now.’ Part of me is just like, what if they just ended up manipulating my mom’s [information] because we had to do background checks, what if they… ratted us out?” (Asian participant) **Food insecurity** “I have a lot of friends that are also freelancers are working like two or three minimum wage… sometimes I will buy my friend a meal when I can because I just got this gig, but other times, I do not know when my next meal is” (Asian participant) “There’s no rice, there’s no beans, there’s no tortillas like everything was just gone… as a graduate student you kind of buy the cheaper stuff like bulk rice and stuff like that… There was a time when they did not even have eggs and so I was like, I do not know what I’m going to buy.” (Latinx participant)
A Climate of Burden: Existing while undocumented	**Learning costs** “I feel like I’ve always been on a flight or fight mode-like I’m on survival mode, actually. And I feel like my parents have always been that way… We’ve always lived in fear… We always have to be extra cautious and make sure we follow the rules by the letter… We’ve always done a really good job at ‘if we are sick, we do like home remedies-we do not seek for professional help’.” (Latinx participant) “So I knew about my status back in elementary school, like pretty early on. My parents wanted me to know that I should not cause trouble to get the police involved… so growing up I never trusted the police because of my immigration status and that just continued on to now.” (Asian participant) **Psychological costs** “I feel that as an immigrant, undocumented person-being a brown, Latina… just physically I feel like I’m a target. I’m a walking target so anything that I do will be used against me… They’re not a symbol of safety for me-they are a threat.” (Latinx participant) “In my break between finishing work and going to class, I thought, ‘Hey, I should, I’m near the beach, like I should go run from work to the beach, and then back to work, get ready go off to class.’ So I decided to throw on a hoodie… within two or three blocks of my workplace… this cop pulls up next to me… and the officer says, ‘You know, you really should not be around this neighborhood anymore.’ He must not have liked what I looked like for that neighborhood and I never went on a run after that-I mean, anywhere.” (Latinx participant) I’m this transition period [of adjusting my immigration status]… you do not want to like screw up anything… So that definitely adds that little like anxiety and fear I guess about what might happen to me if I were to get arrested for any reason and then in the process they turn you into ICE. (Asian participant) **Compliance costs** How am I gonna save up $500 and I heard that they are trying to increase fees on certain things, I do not know if DACA was one of them. It’s like if they increase the fees like how am I gonna do that? You know, on top of what I already have on my plate, you know? (Latinx participant) To cough up 500 bucks [for DACA renewals] each year-that’s a pretty big blow, and having to renew every year means you have to take time off every year to get your biometrics… there’s gonna be a lot more applications… It’s going to slow down the renewal process, because so many people are going at the same time. (Asian participant)
The Impact of Burden in applying for DRAI	**Collective scarcity and individual disempowerment** They [My parents] feel like they cannot take away from others… Like they do not feel the need to take from the government… it was always like “I do not want to take from others if I can, if I can work for it myself… *even under these kinds of circumstances*.” (Latinx participant) “They [My parents] did not know where to get that help or like how to apply for it… they mentioned other people need it more than they do… They did not follow through [in] getting aid.” (Latinx participant) **Fear of Enforcement** “I think one of the first questions asked [about] household income… and that always freaks my parents out because of their immigration status… Even though I think that the format or a disclaimer said something like citizenship is not required. She just was so fearful of whatever consequences might come, it’s not worth the money they try to offer, like, ‘I’ll just scrape by some other way’.” (Latinx participant)
Administrative Burdens in the DRAI Application Process	**Intake Logistics** “You need to know which button you got to hit, you know? And I think there was like, three or two, of those voice recordings. And you need to get through all of them just to, at the end, getting a busy tone on the other side.” (Asian participant) “I was kind of getting anxious about that because first come first serve… I do not know if there’s enough money to go around for everyone. So on the third or fourth day I just had an idea to maybe call right after lunchtime and that’s when they worked… The worker was very helpful, very empathetic. And the process itself took me about like 30 to 45 min. So I can see like how overworked they are. Like one person can take maybe two people at a time, two households at a time per hour, and they work eight hours, that’s only 16 people per day per worker.” (Asian participant) **Language access** “My Tita and I tried the Tagalog application, but you could not get through because I think it wasn’t staffed… she would always get forwarded to voicemail. So she ended up calling the English hotline. Yeah that one was also like, also kind of slammed but we are able to get through eventually.” (Asian participant) “At first I put that in Spanish. And then I wanted to cheat the system and just make it English. I just put English… And then I got through, and I did it for both my parents, but it was like the last week of the program. (Latino participant)
Potential Mediators of Administrative Burden	**The role of young adults** “When you are working with like elders and middle-aged folks, you are just like, wow, I taught someone today how to use zoom that feels like a big accomplishment.” (Asian participant) “I thought I told her something in Korean that I translated it in my head, but the way she understood what I was talking about was very different… we did not receive it [housing grant], but if we did receive it… I’m pretty sure it would have been delayed-I mean there was a whole section that was filled incorrectly.” (Asian participant) **The role of trusted community** “The way that I share information is word of mouth and personal conversations… So now that all these restrictions have been happening… You do not see as many people, you do not talk to as many people, so I just feel there’s people out there missing out on the information, on resources… I’m pretty sure a lot of people did not know about monetary aid.” (Latinx participant) “I think we really rely on building our community on grocery markets! We do actually have all of our livelihoods around one single grocery market. You know, your bulletin board is out there, you meet your community members there, right? And the fact that we aren’t able to do that, I think points to a lot of gaps by how we serve people here.” (Asian participant) “We still check up on each other [online]. It definitely has helped become like a support group, and more of like a community and family. Now more than ever, I think, even though we are like distant, we still try to keep in touch with each other. So it definitely has helped, like, kind of address that need for us to interact with each other.” (Asian participant) **The role of government** “I do not know how I feel about federal right now… I keep it local, so I just look up LA county resources or California, that’s what I trust right now to be honest, the federal government is shady” (Latinx participant). “I feel comfortable. I do not think that they are there to [say], ‘He’s sick, he’s undocumented, he needs to get deported.’ I do not feel like that’s their job. Their job is literally trying to stop the spread, so I do not feel threatened by their existence.” (Latinx participant) “From being undocumented… there’s always that fear that pushes you to manage and try to understand what the situation is really like. The motivation behind the distribution of funding from the governor… there’s likely to be some regulation around it and explicit. It’s a public entity, you are allowed to ask for things, for information so there’s more access… than there would be if a private company comes in… and their role and their intentions and their motivations are completely vague. They’re not visible and the process under which they undergo is actually completely set up by them. It’s much more ambiguity, much less transparency.” (Latinx participant)

Eight participants stated that housing was also a major economic concern during the pandemic. While none of the participants experienced being unhoused at the time of the study, many had to engage in several strategies to remain stably housed, including moving in with family and tapping into savings. Families with undocumented members were also especially vulnerable to housing insecurity in regards to the variable discretion of landlords, as one participant states, “We had heard that apartment complexes, some of them had their landlords have had forgiven the rent for the month. We also had apartment complexes where a couple wasn’t able to pay and the landlord kicked them out.” (Latinx participant) Despite eviction moratoriums in place at both the federal and local levels, another participant highlighted how her mother’s immigration status disempowered her from advocating for her family’s housing rights (Table 4A.1). This participant essentially had to choose between her family’s housing rights and possible deportation. In this case, risking immigration enforcement did not outweigh the economic burden of housing insecurity, especially given her concerns over her landlord’s expressed anti-immigrant political views (Table 4A.1).

A quarter of participants (*N* = 5) also reported experiencing food insecurity during the pandemic as result of both economic hardship as well as from the shortage of supplies early in the pandemic (Table 4A.2).

### A climate of burden: the learning, psychological, and compliance costs of existing while undocumented

Several participants spoke to the experience of being undocumented through the lens of survival. This survival mentality was rooted in abiding by an exhaustive regime of policy that dictates how one could exist to avoid deportation. Even before the COVID-19 pandemic, participants strived to avoid being perceived as a burden to the system to avoid enforcement. The learning costs involve participants understanding from a young age that because of certain policies, accessing basic and fundamental resources, such as healthcare and safety, are always in proximity of deportation (Table 4B.1). Participants then experience the psychological costs of these polices as the fear and stigma that access to services were not meant for them in addition to being criminalized for simply existing (Table 4B.2). This regime of rules creates an atmosphere of vigilance that sustains itself on daily self-surveillance. Participants must constantly evaluate the risk of deportation in taking any given course of action, resulting in an atmosphere in which everyday life is exposed to the threat of criminalization and enforcement (Table 4B.2). Despite California being a well-established sanctuary state since 2017, with explicit policies that limit cooperation between law enforcement and immigration enforcement, half of participants (*N* = 10) reported that the presence of law enforcement generated anxiety and discomfort out of fear of immigration enforcement activity. In particular, Latinx participants reported fears and experiences of being profiled by law enforcement at the intersection of race and immigration status (Table 4B.2). Ultimately, the compliance costs result in individuals forgoing healthcare or avoiding safety services when they may be needed (Tables 4B.1, 2).

Compliance costs can also be manufactured through arbitrary and seemingly punitive rule changes. One example was how the United States Citizenship and Immigration Services (USCIS) announced that DACA would be renewed on a yearly basis instead of every 2 years starting in August 2020. This policy change essentially doubled the renewal cost for DACA as the previous biennial $500 filing fee became an annual payment. In addition to increased monetary costs, individuals may have to take time off from work or school to complete renewal documentation and obtain biometric information collection, which also requires additional fees. Participants affected were keen to highlight the immediate and individual challenges as well as the future structural burdens this change would generate (Table 4B.3). However, in order to maintain their DACA status and avoid deportation, participants could only comply with these changes despite increased burden.

### The impact of burden in applying for DRAI

Participants noted several barriers to applying for DRAI relief that arose in relationship to the larger climate of burden for those who are undocumented. Over a third of participants (*n* = 7) stated that the primary barrier to their families applying for aid was the self-minimization of need that often dovetailed with guilt around taking away resources from others. Perceptions of communal scarcity and collective caring created an emotional nexus of psychological costs that deterred people from participating in DRAI (Table 4C.1). Stigma toward receiving government-based aid was also cited as a psychological cost. Other participants noted that their families’ stated lack of need for the DRAI program veiled a sense of disempowerment in the ability to navigate the process of applying for aid, reflecting high levels of psychological and compliance costs (Table 4C.1).

Three participants reported that the psychological cost of fear of immigration enforcement was the primary barrier to applying for DRAI. One participant had heard of DRAI, but because their parents’ fear to have their “status out in the open” and “actually in paper” was so strong they could not even broach the conversation with them (Asian participant). Another participant did try to convince their mother to apply for DRAI, but they remained fearful of enforcement even though they lived in Los Angeles, which has been a well-established sanctuary city (Table 4C.2). For the third participant, their father’s prior deportation haunted their mother too much for her to consider applying for DRAI. These cases demonstrated how fear, as a psychological cost, can also compound on learning costs, in deterring individuals from even seeking out information on potentially supportive policies and programs.

### Administrative burdens in the DRAI application process

Administrative burden, as an individual experience of policy implementation, was a common experience among participants who applied for DRAI. Challenges included long waiting times and multiple attempts required to complete phone intake due to persistently busy lines, resulting in increasingly high compliance costs of time and resources. One participant noted by the time she was able to get through and complete her intake, she and her mother had collectively logged 900 calls. Others attempted to time their calls to increase their chances for success in getting through (Table 4D.1). A central bottleneck in the process appeared to be the lack of capacity to accommodate a disparately high volume of callers combined with an intake process that required document review, especially as individual CBOs were set up to be fund administrators for entire multi-county regions of California (Table 4D.1). Participants reported calling multiple times from multiple lines, which also added to the strain of intake.

Many participants also felt that limited telephone intake capacity exacerbated issues around gaps in language access. A participant who felt that their parents with limited English proficiency would not be able to navigate the automated telephone system, stated, “You need to know which button you got to hit, you know? And I think there was three or two of those voice recordings and you need to get through all of them just to, at the end, get a busy tone on the other side.” (Asian participant) Participants indicated that some non-English language lines, such as Tagalog, were not well-staffed whereas other language lines, such as Spanish, were oversaturated (Table 4D.2). This led to participants feeling like they needed to “cheat” the system to mitigate compliance costs by dialing the English intake line instead of their preferred language option (Table 4D.2).

### Potential facilitators of administrative burden relief

Participants also discussed several potential factors that can reduce administrative burden in the process of accessing financial aid for undocumented immigrants.

#### Roles of young adults

Participants were key support figures to other members of their households, notably parents and older relatives, across technology and language access issues (Table 4E.1). Having protective status, such as DACA, was a central source of empowerment in navigating through a climate of burden. One participant reflects that knowing they have rights as an immigrant was an important form of liberation that has given them “the guts to get on a plane, talk to police officers” and the “mental freedom to do things” in a way that their parents are not able (Latinx participant). As young adults and digital natives, participants were able to bridge basic technological gaps, such as writing emails for document review (Table 4E.1). However, having to translate and interpret technical content remained a challenge for some and led to missed opportunities. Some participants also stated that undertaking this support role also caused stress within family dynamics, particularly when it comes to serving as a source of information amidst a background of fear and misinformation (Table 4E.1).

### The role of community, community-based organizations, and government

All participants spoke to broad sense of community that served as an important source of support during the pandemic, which included siblings, friends, colleagues, church members. For some participants, the relative isolation social distancing and pandemic changes brought on further highlighted the importance of in-person interactions and community hubs (Table 4E.2). However, some participants did find the expansion of online community as a positive externality of the pandemic in building and maintaining social networks (Table 4E.2).

Some participants underline the importance of familiarity within a community that helps to build trust and empowerment, which also extends to the roles of community-based organizations (CBOs). A strength noted was that since the funding was administered by CBOs that had already helped provide services lowered the activation energy in applying for aid: “[DRAI administration] was by Catholic Charities and I really love their support. They’ve helped me with my DACA renewal as well.” (Latinx participant).

Participants expressed a variety of perspectives across different levels of governments and departments. Overall, most participants were weary of interactions with the government due to fear of immigration enforcement, but some did report increased trust for local and Californian state governments compared to the federal government (Table 4E.3). These participants also felt less fear in their interactions with certain government agencies, such as the Department of Public Health. In addition, a unique perspective was that government funding created more trust in the process as it would imply greater accountability and transparency for data use (Table 4E.3). While signaling trust in government funded relief endeavors, this participant’s unique perspective also demonstrates the deep inherent distrust that exists for institutions.

## Discussion

This was a qualitative study of undocumented Latinx and Asian adults in California and their perspectives on applying for DRAI aid during the COVID-19 pandemic. DRAI was significant for being an unprecedented state government sponsored program to provide direct financial relief to undocumented people who had been excluded from most forms of direct federal aid. This paper represents one of the first studies to explore the direct experiences of undocumented immigrants in navigating the DRAI program and our findings highlight several important issues facing individuals trying to access this resource including: (1) awareness and engagement with the DRAI program, (2) the economic effects of the COVID-19 pandemic, (3) the climate of burden for undocumented immigrants, (4) the administrative burdens of DRAI application, and (5) potential mediators of reducing administrative burden.

The detrimental impacts of the COVID-19 pandemic on the economic conditions of participants and their families echoed what statewide and national data highlighted across employment, housing, and food insecurity. Others have found that twice as many undocumented workers (38%) live below a “living wage” than citizen workers (18%) (2). It is estimated that undocumented workers were only able to access up to $1700 in pandemic unemployment relief compared to upwards of $35,000 for citizen workers (2). In California alone, employers contributed as much as $236 million in tax contributions to the federal unemployment fund for their undocumented employees, yet these workers remain prohibited from accessing these labor benefits (1). In the absence of policy that will allow undocumented immigrants to claim unemployment benefits or qualify for federal aid, DRAI was one of the few mechanisms for state-based relief during the pandemic and is an important policy intervention to evaluate in efforts to improve the social determinants of health for undocumented immigrants.

Participant experiences in applying for DRAI relief spoke to the need of examining the effects of policy-driven administrative burden on the lives of undocumented immigrants and examine how the decision to participate in programs can be impacted by restrictive immigration policies, increased enforcement, and growing anti-immigrant sentiment (9–11, 13). Even after the end of the Trump administration, a 2021 nationwide survey reported that 51% of Latinx immigrants worry that they or someone they know could be deported (21). While larger-scale policy goals pursued during the Trump administration included the reversal of several federal immigration programs, such as DACA and Temporary Protected Status (TPS), the Yale Immigration Policy Tracking Project has shown that out of 1,040 immigration actions created between 2017 and 2020 under the Trump Administration 719 still remain fully in effect across formal and less formal policy modalities, including internal memorandums and bulletins (15).

Over time these policies have coalesced to form an entire regime of burden diffusely embedded across government and society. Moynihan et al. argue that “while any individual action of the Trump administration might be justifiable in isolation, the cumulative pattern of using burdens as a method of exclusion is unmistakable”(8). Furthermore, utilizing regimes as the unit of policy analysis can reveal “the sheer weight that comes with an accumulation of burdens: while any particular barrier might be overcome, the system as a whole becomes increasingly impenetrable-slower, more expensive, demanding, and bewildering”(8). Participants highlighted the example of recent DACA rule changes that increased application frequency and costs. Although the United States Supreme Court ruled in 2020 that DACA was unconstitutionally terminated, policies were simply reconfigured to continue to increase administrative burden by raising financial costs without providing commensurate administrative support for applicants. This process demonstrates how administrative burdens can be deployed in a non-legislative, procedural, and seemingly apolitical manner as a form of “policymaking by other means”(14).

Administrative burden is a fundamental experience of growing up for undocumented young adults. From a young age participants shared how they had a deep understanding of the precarity that policy generated around their immigration status. The learning, psychological, and compliance costs associated with restrictive immigration policy and anti-immigrant sentiment contribute to a vicious cycle of disempowerment, avoiding care, and forgoing services. In regards to applying for relief programs, such as DRAI, this regime of administrative burden directly impacts the choice and ability of immigrants to apply for aid in negotiating with power, risk, and need. Administrative burden displaces the responsibility and onus of service and resource provision from the state to the individual.

The implementation of the DRAI program itself also generated its own set of administrative burdens stemming from the inherent structural constraints of having a 150,000 person limit for an estimated pool of up to 1.6 million potential applicants (5). In regions, such as the California Bay Area, which includes six counties, one CBO was responsible for providing intake for an expected allotment of 30,000 applicants (4). Participant reports of waiting prohibitive amounts of time on busy phone lines or having to make multiple attempts to call before reaching a live person were similar to reports in the media, with the New York Times reporting, for example, that a Los Angeles CBO had 630,000 calls in the first 90 min of the program (6). Participants and their families deployed different counter measures, such as timing calls as well as utilizing English lines to bypass saturation of non-English language lines. While these strategies highlight resourcefulness in successfully accessing resources, they also reflect how immigrants are more vulnerable to administrative burden at the intersections of race, language, and immigration status. As opposed to established entitlement benefits, DRAI was both time and funding limited, creating a system of scarcity, crowding, and anxiety, which led some to experience this relief programs as a high stakes lottery instead.

However, the DRAI program was unique and innovative in how it leveraged several established community-based organizations in administering statewide aid on a regional level. Recognition of trusted community organizations may also reduce longstanding mistrust in government resources. However, it is precisely because of these connections that there may be more at stake for partnering CBOs. While positive interactions can build deeper connections with community, negative interactions may erode that hard-earned trust. While the DRAI program should be considered a collaboration between the state government and regional CBOs, participants described process challenges largely as reflections of individual CBO deficiencies, which may overlook the role and responsibilities that state government support may have had on CBO capacity. There is not currently much publicly available information as to how the CDSS selected the 12 organizations that disbursed aid and what support, technical assistance, and financial resources were provided to each respective CBO to help ensure a more equitable and accessible distribution process.

Many participants, as young adults, served as social brokers to their families and community members in helping to bridge the knowledge, language, and technology gaps in navigating the process of applying for aid (22). It will be important for policy makers and community-based organizations to message toward young adults in addition to their families in future relief efforts as they have potential to mediate burden in applying for aid. However, these efforts need to be undertaken with parallel efforts to provider further administrative support so that burden is not simply shifted on to the shoulders of young people, who also face their own set of challenges.

There is an important research and policy opportunity in better understanding how to facilitate successful collaborations between government and CBOs to provide services and resources for marginalized communities, especially as more opportunities become available to undocumented immigrants, such as the upcoming 2024 expansion of Medicaid in California to all adults regardless of immigration status (23). Policymakers should work to address and mitigate different facets of administrative burden by identifying and including key stakeholders to collaborate on equitable program development and design. Governmental agencies should also work with CBOs to coordinate program awareness across both social and traditional ethnic media networks with special attention given to language access as well as to safety and confidentiality-oriented messaging to address fears of enforcement. Partnerships between CBOs can also be leveraged to help mitigate mistrust of governmental institutions and build trust in public programs to decrease barriers to uptake. To address the dearth of data in regards to government and CBO partnerships, programmatic reports should be published for transparency, program measurement, and process improvement.

Ultimately, the administrative burden that underlies the challenges with implementing relief programs, such as DRAI, is rooted in the policy-driven precarity of being undocumented. Some have suggested solutions outside of immigration policy, such as the amending Affordable Care Act (ACA) to no longer exclude individuals with DACA from the insurance exchanges as a form of “healthcare citizenship” as a stop gap (24). However, even impactful policies, such as DACA, despite evidence of improved educational, economic, and mental health outcomes, have only been able to provide short-term solutions without guarantees for longer-term futures (25–28). Arguably, efforts to eliminate administrative burdens that prevent immigrants from accessing services or resources needed to thrive must address establishing pathways to permanent citizenship, which affords the right to vote and protection against deportation. Given the lack of comprehensive immigration reform, our study identifies programmatic areas that can be addressed to improve program development and implementation in order to mitigate administrative burdens for programs intended to support undocumented immigrants and their families.

This study is limited due the qualitative nature of the data and small sample size. Our aim was not to generalize our participants’ experiences to the entire population of undocumented immigrants, but to highlight that even for a cohort of highly educated, English proficient individuals, administrative burden stemming from immigration status can still be difficult to navigate. While we were not able to directly highlight the voices of undocumented older adults, who remain some of the most vulnerable individuals, our participants’ experiences are helpful proxies that reflect the experiences of populations that have been harder to reach by traditional recruitment methodologies.

Ultimately, further research is needed to better understand the individual experiences of each CBO to help identify and address the regional nuances in regards to outreach, messaging, language access, and capacity development in undertaking the implementation of programs like DRAI. The aim of this work is to neither disparage nor diminish the enormous task undertaken by the state and respective CBOs, but rather to recognize that further improvements are required to better address the social determinants of health for undocumented immigrant communities already stretched thin.

## Data availability statement

The datasets presented in this article are not readily available because of participant confidentiality related to the sensitive nature of our study participants’ immigration statuses. Requests to access the datasets should be directed to MS at msudhinaraset@ucla.edu.

## Ethics statement

The studies involving humans were approved by UCLA Institutional Review Board. The studies were conducted in accordance with the local legislation and institutional requirements. The ethics committee/institutional review board waived the requirement of written informed consent for participation from the participants or the participants’ legal guardians/next of kin because Given the sensitive nature of participants’ immigration status, study team opted for verbal informed consent as to not collect directly identifying information.

## Author contributions

IL: Conceptualization, Formal analysis, Writing – original draft, Writing – review & editing. HC: Data curation, Investigation, Writing – original draft, Writing – review & editing. MS: Conceptualization, Funding acquisition, Investigation, Methodology, Project administration, Resources, Supervision, Writing – review & editing.
